# Agreement of Pain Assessment Using the Short Form of the Canine Glasgow Composite Measure Pain Scale between Veterinary Students, Veterinary Nurses, Veterinary Surgeons, and ECVAA-Diplomates

**DOI:** 10.3390/ani14162310

**Published:** 2024-08-08

**Authors:** Mireia Marco-Martorell, Natalie Duffy, Miguel Martinez, Thomas Maddox, Katherine Robson

**Affiliations:** 1 Leahurst Campus, University of Liverpool, Chester High Road, Neston CH64 7TE, UK; tommad@liverpool.ac.uk (T.M.); katherine.robson@liverpool.ac.uk (K.R.); 2 Northwest Veterinary Specialists, Linnaeus Veterinary Limited, Ashville Point, Beechwood, Sutton Weaver, Runcorn WA7 3FW, UK; natalie.duffy@nwspecialists.com; 3 Chester Gates Veterinary Specialists (CVS), Chester CH1 6LT, UK; mamfer2000@hotmail.com

**Keywords:** dogs, CMPS-SF, agreement, pain, veterinary students

## Abstract

**Simple Summary:**

Pain scoring in dogs can be challenging, particularly in a busy clinical setting and when staff with diverse training and veterinary students are involved. Consequently, concerns about dogs receiving inadequate analgesia were raised at this institution. This study was performed to investigate if veterinary students, veterinary nurses, veterinary surgeons with no specific training in anaesthesia, and diplomates in veterinary anaesthesia and analgesia perform pain scoring using the short form of the canine Glasgow Composite Measure Pain Scale (CMPS-SF) in a similar manner. The results obtained indicated good to excellent pain assessment agreement between groups. Nevertheless, the overall agreement amongst all assessors was poor and the intra-group agreement was poor to moderate, suggesting significant individual differences when pain scoring dogs. Veterinary students tend to pain score higher than more experienced assessors.

**Abstract:**

Several pain scoring systems have been validated to measure pain in dogs. However, pain may not be adequately assessed since these tools are associated with high-level inter-observer variation. The aim of this study is to evaluate the agreement of pain assessment using the CMPS-SF between veterinary students, veterinary nurses, veterinary surgeons, and European College of Veterinary Anaesthesia and Analgesia (ECVAA) diplomates. Forty-five client-owned dogs presented to a teaching hospital were enrolled in this prospective, observational study. All dogs were pain-scored in vivo, while a video of the assessment was recorded and subsequently evaluated by twenty assessors, with five per group. Mean scores between groups were compared, and agreement within groups and agreement of the average scores between groups were assessed by calculating the intraclass correlation coefficient (ICC). The intervention point at which dogs were deemed to require additional analgesia was also evaluated. Overall agreement of pain assessment was poor (ICC = 0.494). Nurses had the best inter-observer agreement (ICC = 0.656), followed by ECVAA diplomates (ICC = 0.540), veterinary surgeons (ICC = 0.478), and veterinary students (ICC = 0.432). The best inter-group agreement was between veterinary surgeons and nurses (ICC = 0.951) and between ECVAA diplomates and nurses (ICC = 0.951). Students were more likely to determine that additional analgesia was required compared to other groups. Pain assessment is key for animal welfare, and training in this area should be reinforced to improve consistency.

## 1. Introduction

The Association for the Study of Pain (IASP) defines pain as “an unpleasant sensory and emotional experience associated with, or resembling that associated with, actual or potential tissue damage” [1,2]. Animals cannot communicate pain verbally, but this does not exclude that an animal can experience pain and requires analgesia [1]. The assessment of pain in veterinary patients remains a challenge because of the complexity of this phenomenon.

The recognition and assessment of pain is an integral part of veterinary clinical practise. The prompt recognition and treatment of acute pain can influence many of the following physiological factors: the prevention of acute neurohumoral changes, reduction in inflammatory cytokines, reduction in inflammatory stress, improving haemodynamic stability, the prevention of postoperative complications, and the prevention of chronic pain states. Pain assessment during emergency triage should also be a priority [3].

Pain in veterinary patients has been measured using behavioural observations quantified with simple tools such as the simple descriptive scale (SDS), numerical rating scale (NRS), and the visual analogue scale (VAS) [4]. However, pain may not be adequately captured by the unidimensional nature of these tools, which often are associated with high-level inter-observer variation [4,5]. Several factors, including anxiety, which may be increased in patients post-operatively, can alter the observer’s perception of the degree of pain an animal is experiencing; conversely, pain may increase anxiety in patients [6]. Discrimination between altered behaviour due to the hospital environment and pain may be difficult, particularly with these unidimensional scales.

**Figure 1:**
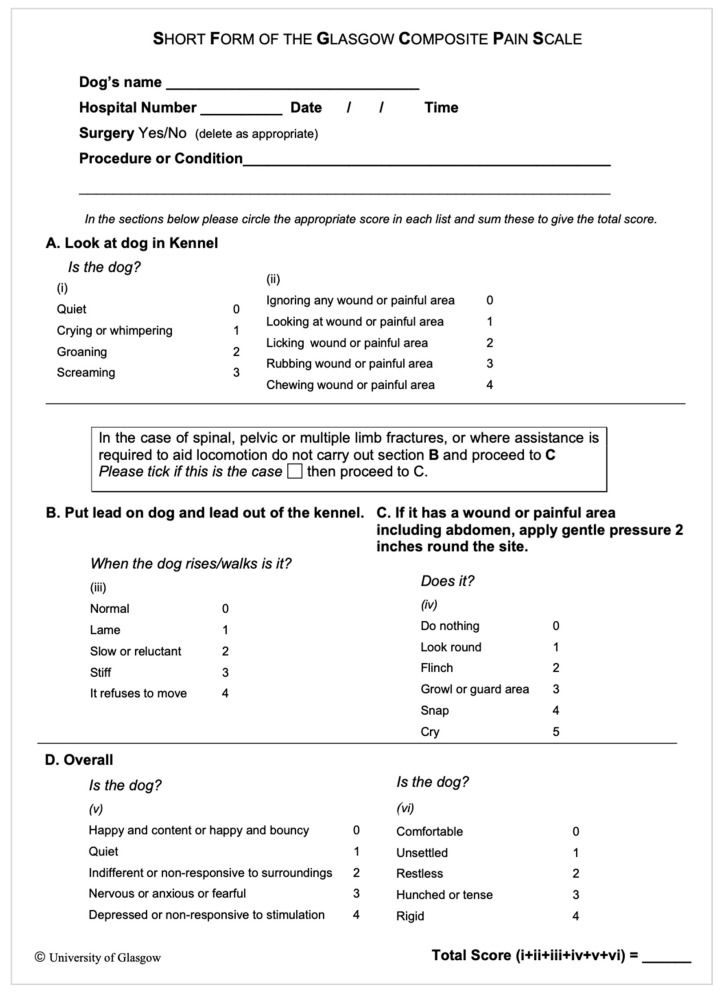
Short form (CMPS-SF) of the Glasgow Composite Measure Pain Scale by [10].

Different evaluation criteria for acute pain using multidimensional scales have also been described in dogs and cats [7]. The Glasgow Composite Measure Pain Scale (CMPS) is a multi-item behavioural pain assessment tool, developed using a psychometric approach, to measure acute pain in dogs [8,9,10]. The short form of this scale (CMPS-SF) (Figure 1) was developed for routine clinical use and involves the completion and scoring of six descriptive categories with associated descriptors (vocalisation, attention to wound, mobility, response to touch, demeanour, and posture/activity) and the assignment of a final score out of 24 if the patient is ambulatory or out of 20 if the patient is non-ambulatory. The CMPS-SF has been validated to measure acute pain in dogs with a defined intervention level for the provision of rescue analgesia [10].

There is evidence in veterinary research of the need for training in pain assessment [11,12]. Research by Barletta [13] found that veterinary students early in their training tend to assign higher pain scores to dogs than experienced veterinary anaesthetists. Another study investigating the inter-observer variability of pain assessment performed by diplomates of the American College of Veterinary Anesthesia and Analgesia (ACVAA) showed no notable differences in pain scores amongst different evaluators [14].

Veterinary surgeons (VSs), registered veterinary nurses (RVNs), and fifth-year veterinary students (VUs) are directly involved in the pain scoring of dogs at the first author’s institution. Although VUs always act under the direct supervision of qualified VSs and/or RVNs, the assessment of pain in veterinary patients remains complex, and concerns were raised by clinicians over patients receiving insufficient analgesic treatment, particularly when this treatment was administered based on pain scores assigned by VUs.

The aim of this study was to evaluate whether VUs, RVNs, VSs (without specific training in pain scoring), and diplomates from the European College of Veterinary Anaesthesia and Analgesia (ECVAA Dip) perform pain assessment using CMPS-SF in a similar manner. We hypothesised that there is agreement between VUs, RVNs, VSs, and ECVAA Dips performing pain scores in dogs. This study also investigated the intervention point at which dogs were deemed to require additional analgesia based on pain scores assigned by different accessors.

## 2. Materials and Methods

### 2.1. Study Design

This prospective, observational study was approved by the University Research Ethics Committee (VREC 1163) of the University of Liverpool. Clients’ written consent was obtained prior to enrolment for all cases. All dogs included in the study were able to ambulate and were deemed to have amenable behaviour. Dogs with unsuitable behaviour and/or conditions impeding the ability to walk were excluded from this study. Behavioural assessment prior to pain scoring was performed by the evaluator (M.M.-M.; see below); this was based on a brief evaluation of the dog’s sociability (assessing how the dog coped during human interaction) and confidence during an unfamiliar procedure such as pain assessment, particularly in the concurrent presence of potential pain. If the dog showed signs of aggression, such as biting attempts, growling, or snapping at the air, or was deemed to be too fearful to allow full pain scoring, it was not selected for further evaluation.

Assuming a moderate intra-class correlation coefficient (ICC) of 0.7 or more, our sample size calculation indicated that, to assess the agreement between pain scores performed by 20 observers (in 4 groups of 5 observers), 45 dogs were required to estimate such ICC with a precision of 10%, a 95% confidence interval, and 80% power. Therefore, based on this power calculation, a total of 45 client-owned dogs were recruited for this study. Case recruitment took place between February and June 2022.

Video recordings were made of each dog undergoing pain assessment using the CMPS-SF. Pain scoring was performed by a single ECVAA resident (M.M-M; evaluator) in dogs following a variety of surgical and non-surgical interventions. All dogs were assessed by the evaluator and video-recorded in the same room under the same circumstances (i.e., hospital wards or intensive care unit) to simulate real-life pain scoring. Filming was performed using a mobile phone device (iPhone X-Apple, Apple, London, United Kingdom 2018) for approximately 60 s. For dogs that were anaesthetised prior to assessment, pain scoring was only performed when the patient was awake, alert, responsive, and ambulatory. During the 60 s assessment, the first 10 s were dedicated to recording the dog in the kennel, maintaining no interaction with the evaluator. After that, the evaluator interacted verbally with the dog for 10 s, using positive reinforcement. The dog’s name was not used during recording to maintain patient confidentiality. Following the evaluator’s introduction to the dog, the kennel door was opened to allow the patient outside, using a lead or harness. Pain scoring was performed using the CMPS-SF; all dogs were walked a few steps and gentle palpation and pressure around the area of interest was performed to assess for signs of discomfort. Analgesic drugs were not withheld for the purpose of this study and were administered based on the Small Animal Teaching Hospital (SATH) standard of care. Methadone 0.2 mg kg^−1^ (Methadyne 10 mg mL^−1^; Jurox Pty Limited, Rutherford, Australia) was intravenously (IV) provided if the pain score was ≥6/24. This study followed the recommended analgesic intervention threshold proposed by the authors of the CMPS-SF scale [10].

To recruit assessors, email invitations containing study information and a consent form were distributed to all VUs, RVNs, VSs, and ECVAA Dips at the SATH between August and September 2022. Participation was voluntary and their participation and results had no impact on the VU final grades. Staff members and students who were first to reply to our study participation email were recruited to the study. Five external ECVAA Dips were also invited to participate in this research study; participation of external specialists was required to achieve an adequate number of participants.

When the assessor consent form was signed and returned to the main investigator (M.M.-M.), another email was sent to each participant (five per group) with a participant number and spreadsheet. All participants were added to a private Microsoft Teams™ 2022 channel, which was linked to the previously recorded videos and a demonstration containing detailed explanations of pain assessment using CMPS-SF. Each video was assigned a code name, and the order of the videos was randomised prior to distribution so that each person within a group viewed the videos in a different order. None of the chosen participants were primarily involved in the care of the dogs enrolled in this study.

### 2.2. Statistical Analysis

Statistical analyses were performed using SPSS statistical software (IBM SPSS Statistics for Windows, Version 27.0. Armonk, NY, USA. The normality of data were assessed by graphical analysis and with the Shapiro–Wilk test. The mean scores for each animal between groups were compared using Related Samples Friedman’s Two-Way Analysis of Variance, followed by the Dunn post hoc test with Bonferroni correction for multiple comparisons. The agreement of all individual scores within groups and the agreement of the mean scores for each animal between groups were assessed by calculating the intraclass correlation coefficient (ICC) with 95% confidence intervals (CI), using a two-way random-effect model (with raters and participants considered random) for absolute agreement. ICC values were evaluated as described by [15].

For the evaluation of the intervention point, which we defined as the point at which an animal is deemed to require additional analgesia based on pain scoring (≥6/24), the binary agreement within groups and between groups was assessed with Fleiss’ Kappa. The binary agreement of the modal response between groups was assessed with Cohen’s Kappa and evaluated as described by [16]. *p* < 0.05 was considered significant for all analyses. The scores for all the participants were converted into binary categories: scores of 6 or greater (out of 24) were recorded as ‘Yes’, the dog required an intervention (i.e., additional analgesia); scores of 5 or lower (out of 24) were recorded as ‘No’, the dog did not require additional analgesia. The binary categories assigned (Yes/No) were based on whether analgesic intervention was required [10].

## 3. Results

### 3.1. Animals

A total of 45 dogs were recruited for the study, including 23 females and 22 males. The median age was 75 months (3–195 months), and the median bodyweight was 21.1 kg (4–43.8 kg). Different breeds were included in the study; crossbreed dogs were the most common *(n* = 18), followed by French bulldog (*n* = 4), Border collie (*n* = 4), Labrador retriever (*n* = 3), Border terrier (*n* = 2), Staffordshire bull terrier (*n* = 2), Schnauzer (*n* = 2), American bulldog (*n* = 1), Beagle (*n* = 1), German shepherd (*n* = 1), Cavalier King Charles Spaniel (*n* = 1), Pug (*n* = 1), Poodle (*n* = 1), Lurcher (*n* = 1), Italian Greyhound (*n* = 1), West highland white terrier (*n* = 1), and Golden retriever (*n* = 1).

Prior to pain scoring, dogs had undergone different surgical procedures or had underlying medical conditions that were expected to be painful. Surgical procedures included the following: tibial plateau levelling osteotomy (TPLO) (*n* = 23); humeral condylar fracture repair (*n* = 4); total hip replacement (THR) (*n* = 3); tibial fracture repair (*n* = 3); tibial tuberosity transfer (TTT) (*n* = 1); partial tarsal arthrodesis explant (TTT) (*n* = 1); ovariohysterectomy (OHT) (*n* = 1); mammary mastectomy (*n* = 1); and mast cell tumour scar excision (*n* = 1). Non–surgical cases included spinal myelopathy (*n* = 5); polyarthritis (*n* = 1); and pancreatitis (*n* = 1).

### 3.2. Participants (Assessors)

A total of twenty participants were recruited and subsequently divided into four groups according to their role: VU, RVN, VS, ECVAA Dip. The assessor group included fourteen females and six males. The age range (in years) varied amongst the different groups as follows: VU (22–25), RVN (25–40), VS (25–40), and ECVAA Dip (30–40).

### 3.3. Group Comparison

**Figure 2:**
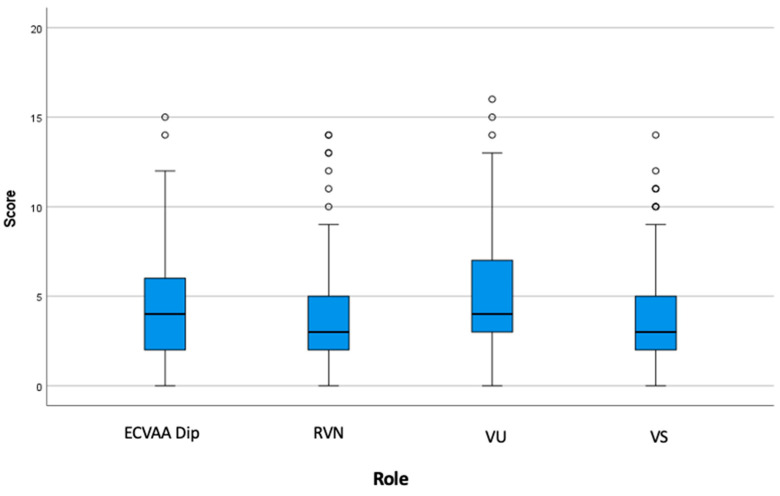
Box and whisker plot summarising all pain scores for each of the groups: ECVAA diplomates (ECVAA Dips), registered veterinary nurses (RVNs), fifth-year veterinary students (VUs), and veterinary surgeons (VSs). Boxes represent the interquartile range from the 25th and 75th percentile. The horizontal bar in each box represents the median value for all scores. The whiskers indicate the range of data values unless outliers are present, in which case the whisker extend to a maximum of 1.5 the interquartile range. Such outlying data points are represented by dots.

The overall agreement of pain assessment (across all assessors) was poor (ICC = 0.494, 95% CI 0.390–0.616) and the VU mean scores were significantly different from VS (*p* < 0.001), RVN (*p* < 0.001), and ECVAA Dip (*p* = 0.048) mean scores (Figure 2 and Table 1). Intra-group agreement was poor to moderate [15]. RVNs had the best intra-group agreement (ICC = 0.656, 95% CI 0.537–0.767), followed by ECVAA Dips (ICC = 0.540, 95% CI 0.407–0.675), VSs (ICC = 0.478, 95% CI 0.339–0.675), and VUs (ICC = 0.432, 95% CI 0.296–0.582).

VU mean scores were significantly higher than the other groups (Table 1), and their agreement with the other groups was the poorest (Table 2). The highest inter-group agreement was between VSs and RVNs (ICC = 0.951 95% CI 0.910–0.973) and between ECVAA Dips and RVNs (ICC = 0.951, 95% CI 0.901–0.975) (Table 2), showing a good to excellent agreement between these groups.

The overall agreement for analgesic intervention (all assessors) was fair (ICC = 0.350, 95% CI 0.327–0.374). The agreement followed the same trend as before; RVNs had the best intra-group agreement (moderate agreement, κ = 0.470, 95% CI 0.361–0.572), followed by ECVAA Dips (fair agreement, κ = 0.385, 95% CI 0.288–0.473), VSs (fair agreement, κ = 0.321 95% CI 0.218–0.430), and VUs (fair agreement, κ = 0.297 95% CI 0.204–0.382). Between groups, VUs and ECVAA Dips and VUs and RVNs had substantial agreement with κ values at 0.695 and 0.663, respectively. The following groups showed moderate agreement: VUs and VSs (κ = 0.604), RVNs and ECVAA Dips (κ = 0.549), and VUs and ECVAA Dips (κ = 0.483). VUs and RVNs had only fair agreement (κ = 0.353). Table 3 illustrates the different analgesic intervention points amongst the different groups of assessors. When looking at the modal answer for each of the groups, all the groups agreed that additional analgesia was required for five of the forty-five dogs and was not required for twenty-six of the dogs. Looking at the individual responses, there was only one dog where every single participant agreed that the animal needed analgesia and only three dogs where every participant agreed that analgesia was not required. VUs were more likely to determine that additional analgesia was required compared to other groups.

## 4. Discussion

To the authors’ knowledge, this is the first study looking at the agreement between veterinary professionals with different roles and levels of expertise when pain scoring dogs in a hospital setting. Our study shows good to excellent inter-group agreement of pain assessment using CMPS-SF, meaning that pain score results are comparable across the four groups that we evaluated (ECVAA diplomates, registered veterinary nurses, fifth year-veterinary students, and veterinary surgeons). Nevertheless, the overall agreement across assessors appears to be poorer and intra-group agreement is poor to moderate, indicating that individuals within the same group often provide disparate scores. This corroborates the idea that pain assessment in veterinary patients remains a challenge, particularly because of the complexity of this subjective phenomenon. Additionally, the observer’s perception of the degree of pain an animal is experiencing is affected by several factors, including behavioural states such as anxiety [5]. Pain sensitivity and perception of pain may also differ based on other factors, such as dog breed; for example, hunting and working dogs have been shown to have a higher pain sensitivity threshold [17]. Therefore, even when using validated pain scales, factors which may interfere with their use should be considered.

Veterinary training in the field of pain assessment should be further expanded to ensure adequate pain scoring and analgesia provision in small animals. Veterinary educational institutions should promote training to ensure that veterinary students are familiar with pain scores and feel comfortable performing pain assessment in veterinary species. Although small animal veterinary surgeons appear to demonstrate awareness of pain in their patients and employ various methods for pain assessment, a limited use of validated tools is identified by some authors [18] and is likely attributed to challenges such as a lack of an established routine, time constraints, insufficient staff, and, particularly, a knowledge gap among veterinary staff who do not employ pain assessment scales routinely. This suggests a window of opportunity for the implementation of training programmes in small animal pain assessment.

Our study was focused on the canine species, but it is important to note that pain assessment differs between species, mainly due to variations in behaviours associated with pain. Species-specific training should be considered to ensure adequacy when assessing pain in different species. The type of pain and pain scale validation should also be considered. In cats, for example, pain assessment can be particularly challenging due to their nature and how they behave in a hospital setting. CMPS has been validated to assess pain in cats (with a final score out of 20 instead of 24 as for dogs) [19], and there are established levels of interventions for the provision of rescue analgesia in feline patients. Facial expression is considered a sensitive indicator of noxious procedures in cats. The assessment of such expressions (Feline Grimace Scale, FGS) improves the performance of behaviour-based tools, as previously described [20]. The FGS is reliable for feline acute pain assessment even when used by assessors with different experience [20].

Based on our results, the overall agreement of pain assessment amongst participants appears to be suboptimal, resulting in wide discrepancies within the same group of assessors. Because of this, the authors suggest that trainees are encouraged to pain score patients in small groups (2–3 people) to optimise comparison and ensure more consistent results among different assessors. Comparison is a powerful learning process that has been leveraged to improve training in a variety of domains [21]. Group pain assessment could potentially improve both inter-group and intra-group agreement. Another area of improvement suggested by the authors is the development of pain scales specific for the condition being assessed (e.g., ocular pain, visceral pain, orthopaedic pain). Condition-specific pain scales are established in human pain assessment [22,23,24].

Regarding the evaluation of agreement over the provision of additional analgesia based on pain scores, our data showed a moderate to fair agreement on the requirement for additional analgesia within the different groups and good to moderate agreement between groups. It appears from our results that the agreement across assessors improves when pain scores are determined to be either high (additional analgesia required) or low (no additional analgesia required). This suggests that, regardless of the discrepancies in pain score results across the different assessors, participants generally appear to agree on the requirement for additional analgesia. Therefore, pain score agreement appears to be more consistent in dogs with either high or low pain scores, in which the need (pain score ≥ 6/24) or lack thereof (pain score ≤ 5/24) for additional analgesia is less questionable.

Our results reveal that VU tend to score pain higher compared with more experienced assessors and, therefore, students are more proactive when it comes to considering additional analgesia based on pain scores. This same observation has been made before by [13]. Other studies have shown that veterinary surgeons with less experience also tend to assign higher pain scores to dogs that are given lower scores by more experienced colleagues [25]. Although the exact reason for this tendency is unclear, it could be that newer generations of veterinary surgoens are more aware of pain and its assessment in practise. Consequently, dogs are unlikely to receive insufficient analgesia when rescue analgesia is delivered based on pain scores proposed by VUs. Nonetheless, it is important to note that the overestimation of pain (suspected amongst those less experienced assessors, including VUs) could possibly result in the overprescription of analgesic drugs, which is not without side effects, particularly when using drugs such as opioids. A retrospective study comparing two analgesic strategies in dogs after TPLO [26] found that dogs receiving methadone every four hours regardless of pain score did not necessarily have a better outcome than those that received methadone based on pain score. In fact, dogs receiving methadone irrespective of pain score were more likely to vomit and vocalise, with a reduction in food intake whilst hospitalised. This emphasises the importance of patients undergoing adequate pain assessment and receiving opioid analgesia only when necessary.

There are several limitations to our study. The first limitation is that pain was evaluated by assessors through pre-recorded videos rather than in a real-life setting. Video-recordings have been used in research for the assessment of the quality of recovery from anaesthesia and postoperative pain [14], and good agreement between video versus real-life is described [27]. Some pain assessors in our study raised concerns related to the inability to differentiate background noise and other sounds such as ‘whining’ that could represent pain and anxiety. Pre-recorded pain assessment videos also precluded direct interactions between the assessors and patient, as is the case in a real-life scenario. Therefore, the authors acknowledge that videos should be used with caution and that pain assessment based on pre-recorded videos may differ from pain assessment in a real-life scenario. Our best effort was put in to recreating a realistic clinical setting. In fact, dogs are usually pain-scored in kennels with background noise from other dogs and veterinary staff. Ultimately, video-recorded pain assessment was deemed to be the only feasible (and ethical) method to evaluate the same dog 20 times for the purposes of this study.

Overall participation (assessor recruitment) was optional. Staff members and students who were first to reply to our study participation email were recruited. This could have created an artificial selection bias in which staff and students with greater interest in pain management and pain scoring were more likely to participate, altering our study results [28]. Furthermore, our assessor group had a female bias (fourteen females versus six males), which could have also influenced pain assessment. Several studies have shown that female participants tend to report pain more frequently, tend to be more empathetic, and are likely to pain score higher compared to males, especially when female assessors experience chronic pain conditions themselves [29,30]. In a study performed in cats, female veterinarians were more likely to assign higher pain scores and administer analgesics than male individuals [31,32]. Multiple biopsychological mechanisms may account for such sex differences when performing pain scoring as follows: sex hormones, endogenous opioid function, genetic factors, pain coping strategies, and gender roles [29]. For example, pain sensitivity appears to change across the menstrual cycle, with increased sensitivity during the luteal phase [30] Nevertheless, in our study, despite the aforementioned considerations, the female bias within the assessor group reflected the reality of our institution and the wider profession in which pain assessment is more likely to be carried out by female assessors.

## 5. Conclusions

Our study shows good to excellent inter-group agreement of pain assessment using CMPS-SF. Nevertheless, the overall agreement across different pain assessors appears to be suboptimal, resulting in poor to moderate intra-group agreement. Our data also show that veterinary students tend to pain score higher and have poorer inter-individual agreement compared to more experienced assessors.

Our findings support the idea that the assessment of pain in veterinary patients remains a challenge, particularly because of the complexity of this subjective phenomenon. Pain assessment is key to ensure that animal welfare and training in this area is reinforced to improve consistency. CMPS-SF is a validated tool, but further refinement for specific clinical scenarios may be needed to also improve consistency.

## Figures and Tables

**Table 1:** Summary of the mean pain scores given by each group for the 45 dogs evaluated by four groups of observers. *p*-values are from the Related Samples Friedman’s Two-Way Analysis of Variance, followed by the Dunn post hoc test with Bonferroni correction for multiple comparisons.

Group	Median of Mean Scores	Interquartile Range (IQR)	*p* Value (Ref. Veterinary Students)
Fifth-year veterinary students	4.6	3.2–6.4	
Registered veterinary nurses	3.4	2.5–4.7	*p* < 0.001
Veterinary surgeons	3.6	2.4–4.5	*p* < 0.001
ECVAA diplomates	3.6	2.7–5.7	*p* = 0.048

**Table 2:** Inter-group agreement of pain scores. VUs, fifth-year veterinary students; RVNs, registered veterinary nurses; VSs, veterinary surgeons; ECVAA Dips, ECVAA diplomates; ICC, intraclass correlation coefficient; 95% CI, 95% confidence intervals.

Group	ICC	95% CI
VU vs. RVN	0.824	0.427–0.927
VU vs. VS	0.819	0.417–0.924
VU vs. ECVAA Dip	0.840	0.653–0.920
RVNs vs. VS	0.951	0.910–0.973
RVNs vs. ECVAA Dip	0.951	0.901–0.975
VS vs. ECVAA Dip	0.928	0.864–0.961

**Table 3:** Agreement between groups regarding whether additional analgesic intervention required. The modal response for each group is presented as follows: + (Yes) Provide analgesia (Pain score ≥ 6/24); - (No) No need for additional analgesia (Pain score ≤ 5/24). Vus, fifth-year veterinary students; RVNs, registered veterinary nurses; VSs, veterinary surgeons; ECVAA Dips, ECVAA diplomates.

	Modal Answer			
ROLE	VU	RVN	VS	ECVAA Dip
DOG 1	+	-	-	-
DOG 2	+	+	+	+
DOG 3	+	+	+	+
DOG 4	+	-	-	-
DOG 5	+	-	-	-
DOG 6	-	-	-	-
DOG 7	-	-	-	-
DOG 8	-	-	-	-
DOG 9	-	-	-	-
DOG 10	-	-	-	-
DOG 11	-	-	-	-
DOG 12	-	-	-	-
DOG 13	-	-	-	-
DOG 14	+	-	-	-
DOG 15	+	-	-	-
DOG 16	+	-	+	+
DOG 17	-	+	-	+
DOG 18	-	-	-	-
DOG 19	+	-	-	-
DOG 20	+	-	+	+
DOG 21	-	-	-	-
DOG 22	-	-	-	-
DOG 23	-	-	-	-
DOG 24	-	-	-	-
DOG 25	+	-	-	-
DOG 26	+	+	+	+
DOG 27	+	+	+	+
DOG 28	+	+	+	+
DOG 29	-	-	-	-
DOG 30	-	-	-	-
DOG 31	-	-	-	-
DOG 32	-	-	-	-
DOG 33	-	-	-	-
DOG 34	-	-	-	+
DOG 35	-	-	-	-
DOG 36	-	-	-	-
DOG 37	-	-	-	-
DOG 38	+	-	+	+
DOG 39	-	-	-	-
DOG 40	-	-	-	-
DOG 41	-	-	-	-
DOG 42	-	-	-	-
DOG 43	-	-	-	-
DOG 44	-	-	-	+
DOG 45	-	-	-	-
Analgesic Intervention Required (Yes)	15	6	8	13

## Data Availability

Data are contained within the article.

## References

[1] Jarrel (1979). Pain terms: A list with definitions and notes on usage. Recommended by the IASP Subcommittee on Taxonomy. Pain.

[2] Raja S.N., Carr D.B., Cohen M., Finnerup N.B., Flor H., Gibson S., Keefe F.J., Mogil J.S., Ringkamp M., Sluka K.A. (2020). The revised International Association for the Study of Pain definition of pain: Concepts, challenges, and compromises. Pain.

[3] Rousseau-Blass F., O’Toole E., Marcoux J., Pang D.S.J. (2020). Prevalence and management of pain in dogs in the emergency service of a veterinary teaching hospital. Can. Vet. J..

[4] Holton L.L., Scott E.M., Nolan A.M., Reid J., Welsh E. (1998). Relationship between physiological factors and clinical pain in dogs scored using a numerical rating scale. J. Small Anim. Pract..

[5] Holton L.L., Scott E.M., Nolan A.M., Reid J., Welsh E., Flaherty D. (1998). Comparison of three methods used for assessment of pain in dogs. J. Am. Vet. Med. Assoc..

[6] Ellwood B., Murison P.J. (2022). Investigating the effect of anxiety on pain scores in dogs. Vet. Anaesth. Analg..

[7] Hernandez-Avalos I., Mota-Rojas D., Mora-Medina P., Martínez-Burnes J., Casas Alvarado A., Verduzco-Mendoza A., Lezama-García K., Olmos-Hernandez A. (2019). Review of different methods used for clinical recognition and assessment of pain in dogs and cats. Int. J. Vet. Sci. Med..

[8] Holton L., Reid J., Scott E.M., Pawson P., Nolan A. (2001). Development of a behaviour-based scale to measure acute pain in dogs. Vet. Rec..

[9] Morton C.M., Reid J., Scott E.M., Holton L.L., Nolan A.M. (2005). Application of a scaling model to establish and validate an interval level pain scale for assessment of acute pain in dogs. Am. J. Vet. Res..

[10] Reid J., Nolan A., Hughes J., Lascelles D., Pawson P., Scott E. (2007). Development of the short-form Glasgow Composite Measure Pain Scale (CMPS-SF) and derivation of an analgesic intervention score. Anim. Welf..

[11] Murinson B., Mezei L., Nenortas E. (2011). Integrating cognitive and affective dimensions of pain experience into health professions education. Pain. Res. Manag..

[12] Yanni L.M., Priestley J.W., Schlesinger J.B., Ketchum J.M., Johnson B.A., Harrington S.E. (2009). Development of a comprehensive e-learning resource in pain management. Pain. Med..

[13] Barletta M., Young C.N., Quandt J.E., Hofmeister E.H. (2016). Agreement between veterinary students and anesthesiologists regarding postoperative pain assessment in dogs. Vet. Anaesth. Analg..

[14] Hofmeister E.H., Barletta M., Shepard M., Brainard B.M., Trim C.M., Quandt J. (2018). Agreement among anesthesiologists regarding postoperative pain assessment in dogs. Vet. Anaesth. Analg..

[15] Koo T.K., Li M.Y. (2016). A Guideline of Selecting and Reporting Intraclass Correlation Coefficients for Reliability Research. J. Chiropr. Med..

[16] Landis J.R., Koch G.G. (1977). The measurement of observer agreement for categorical data. Biometrics.

[17] Caddiell R.M.P., Cunningham R.M., White P.A., Lascelles B.D.X., Gruen M.E. (2023). Pain sensitivity differs between dog breeds but not in the way veterinarians believe. Front. Pain Res..

[18] Menéndez S., Cabezas M.A., Gomez de Segura I.A. (2023). Attitudes to acute pain and the use of pain assessment scales among Spanish small animal veterinarians. Front. Vet. Sci..

[19] Reid J., Scott E.M., Calvo G., Nolan A.M. (2017). Definitive Glasgow acute pain scale for cats: Validation and intervention level. Vet. Rec..

[20] Evangelista M.C., Watanabe R., Leung V.S.Y., Monteiro B.P., O’Toole E., Pang D.S.J., Steagall P.V. (2019). Facial expressions of pain in cats: The development and validation of a Feline Grimace Scale. Sci. Rep..

[21] Rittle-Johnson B., Star J. (2011). The power of comparison in learning and instructions: Learning outcomes supported by different types of comparisons. Psychol. Learn. Motiv..

[22] Caudle L.E., Williams K.A., Pesudovs K. (2007). The Eye Sensation Scale: An ophthalmic pain severity measure. Optom. Vis. Sci..

[23] Trninić Z., Spahalić M., Galić G., Kozomara D., Lasić V., Bevanda D., Šutalo N. (2017). Pain Intensity Scales Comparison in Patient with Abdominal Pain. Psychiatr. Danub..

[24] Aydin F., Aksit E., Yildirim O.T., Aydin A.H., Dagtekin E., Samsa M. (2019). Chest pain score: A novel and practical approach to angina pectoris. A diagnostic accuracy study. Sao Paulo Med. J..

[25] Caraceni A., Shkodra M. (2019). Cancer Pain Assessment and Classification. Cancers.

[26] Kongara K., Squance H.E., Topham I.A., Bridges J.P. (2016). Attitudes and perceptions of veterinary paraprofessionals in New Zealand to postoperative pain in dogs and cats. N. Z. Vet. J..

[27] Bini G., Vettorato E., De Gennaro C., Corletto F. (2018). A retrospective comparison of two analgesic strategies after uncomplicated tibial plateau levelling osteotomy in dogs. Vet. Anaesth. Analg..

[28] Catanzaro A., Di Salvo A., Steagall P.V., Zampini D., Polisca A., Della Rocca G. (2016). Preliminary study on attitudes, opinions and knowledge of Italian veterinarians with regard to abdominal visceral pain in dogs. Vet. Anaesth. Analg..

[29] Doodnaught G.M., Benito J., Monteiro B.P., Beauchamp G., Grasso S.C., Steagall P.V. (2017). Agreement among undergraduate and graduate veterinary students and veterinary anesthesiologists on pain assessment in cats and dogs: A preliminary study. Can. Vet. J..

[30] Bartley E.J., Fillingim R.B. (2013). Sex differences in pain: A brief review of clinical and experimental findings. Br. J. Anaesth..

[31] Christov-Moore L., Simpson E.A., Coudé G., Grigaityte K., Iacoboni M., Ferrari P.F. (2014). Empathy: Gender effects in brain and behavior. Neurosci. Biobehav. Rev..

[32] Evangelista M., Steagall P. (2021). Agreement and reliability of the Feline Grimace Scale among cat owners, veterinarians, veterinary students and nurses. Sci. Rep..

